# Supplementary material for: Behaviour and vocalizations of two sperm whales (Physeter macrocephalus) entangled in illegal driftnets in the Mediterranean Sea
Source: PLoS One. 2021 Apr 29;16(4):e0250888. doi: 10.1371/journal.pone.0250888 (PMC8084192; doi:10.1371/journal.pone.0250888)
Supplement: Attachment — Submitted filename: Response to Reviewers.docx [file pone.0250888.s002.docx]

**AR in this file is Authors Reply**

**Response to Editor’s comments**

Journal Requirements:

Please review your reference list to ensure that it is complete and correct. If you have cited papers that have been retracted, please include the rationale for doing so in the manuscript text, or remove these references and replace them with relevant current references. Any changes to the reference list should be mentioned in the rebuttal letter that accompanies your revised manuscript. If you need to cite a retracted article, indicate the article’s retracted status in the References list and also include a citation and full reference for the retraction notice.

**AR: we rechecked all the reference list and corrected eventual formatting errors, in accordance with PLOSONE guidelines.**

Additional Editor Comments (if provided):

You have addressed positively the substantial suggestions of the reviewers. However, I find that the article still contains errors and one unsupported claim that should be de-emphasized in your manuscript. I am therefore providing herein a detailed list of changes required for the manuscript to be acceptable, both in terms of content and presentation.

Major content change. Your observations were important, but limited. There is not sufficient scientific content in your report to ascribe reasons for outcomes in the two release attempt scenarios. While it might be that the presence of more boats was a factor causing higher responsiveness in the second whale, you cannot test that scientifically. Therefore, I suggest you cut the final sentence from the abstract, and significantly shorten the “Recommendations” section related to guidance of rescue protocols. I suggest any recommendations you state here should follow those of the Hamer and Minton publication. For example, on pg 13, those authors state “a quieter environment (less situational stress)… will increase the likelihood of survival…”.

Therefore, I suggest the recommendations section be cut down to a simple paragraph in which you emphasize guidance already published (citing the source) as much as possible. The one specific contribution your study makes is the benefit of including a hydrophone to observe the sounds of the whales during release attempts, as these might be helpful to interpret the state of the animal during operations.

**AR:**

The other detailed suggestions below are based upon my detailed review of the manuscript. I hope you will be able to accept them all.

**AR: thank you for your suggestions, we agreed with them all.**

Ln 26: change ‘provide’ to ‘collect’

**AR: changed**

Ln 27: change ‘notably’ to ‘likely’

**AR: accepted**

Lns 31, 32, and throughout: change ‘breath rate’ to ‘breathing rate’

**AR: accepted**

Ln 31 – report here the observed breathing rate for the first animal

**AR: added**

Ln 32 – report here the observed breathing rate for the second animal

**AR: added**

Ln 36 – after ‘single clicks’ insert “ – likely either slow clicks or regular clicks” ((this is necessary as ‘single click’ is not an accepted form of click type in the sperm whale literature, as pointed out by reviewer 3))

**AR: added**

Lns 36-37: change “By comparing all data it was found that” to “Our observations indicate that”

**AR: changed**

Ln 37: change “animal’s physical/physiological status” to “physical/physiological status of sperm whales” -- this is the key scientific contribution of this report.

**AR: changed**

Delete the last sentence in the abstract, which is 1) controversial and 2) not scientifically supported by the paper. This sort of suggestion should only be mentioned in the discussion where some ancillary material is acceptable. I suggest you use positive wording for this point.

**AR: deleted**

Ls 45 – change “Atlantic one” to “Atlantic population”

**AR: changed**

Ln 46 – change ‘this population’ to ‘the Mediterranean’

**AR: changed**

Ln 48 – insert “a” before “concerning matter”

**AR: added**

Ln 48 – interest ‘the’ before “international”

**AR: added**

Ln 65 – change ‘makes’ to ‘is’

**AR: changed**

Ln 74 – add ‘as sounds could not be ascribed to individual whales’ after ‘social unit’

**AR: added**

Ln 77 – add ‘production’ after ‘acoustic’

**AR: added**

Lns 79-80 – delete ‘intense’ before ‘stressful’

**AR: added**

Lns 80 – 86 delete these two sentences here, as they belong in the discussion. The text to this point provides adequate motivation and background.

**AR: deleted**

Ln 96 – change ‘shown’ to ‘had’

**AR: changed**

Ln 99 – change ‘Genre’ to ‘Sex’

**AR: changed**

Ln 105 – use past tense to refer to your specific study results, so change ‘are’ to ‘were’

**AR: changed**

Ln 106 – change ‘are described as follow’ to ‘were’

**AR: changed**

Ln 108 – change ‘specific phases for’ to ‘phases specific to’

**AR: changed**

Ln 115 – change ‘has been observed, which was described as’ to ‘was observed:’

**AR: changed**

Ln 117 – change ‘has been’ to ‘were’

**AR: changed**

Ln 123 – change ‘has been’ to ‘was’

**AR: changed**

Ln 129 – change ‘percentage’ to ‘rate’ (this is number per unit time). It should be reported as a number per unit time

**AR: changed**

Ln 136 – delete ‘mainly’

**AR: done**

Ln 149 – delete ‘for’

**AR: done**

Lns 149-150 change ‘for “single clicks” are intended sequences of clicks (ICI > 1 s), not organized in a defined structure, similar to ‘usual click’ described in literature [39]’ to “single clicks” were scored for sequences of clicks (ICI > 1 s) that were not organized in a defined structure, as described for ‘usual clicks’ [39]’

**AR: changed**

Ln 154 – delete ‘post-hoc’

**AR: done**

Ln 172 – state here the date the second whale was sighted

**AR: done**

Ln 176 – add ‘for small cetaceans’ after ‘recommended’

**AR: added**

Ln 192 – delete ‘of which’

**AR: deleted**

Ln 193 and Figure 4 – the methods describe this as the number of events divided by the total number of sessions. Therefore, this is a rate. The Y-axes should be number of events per unit time.

**AR: changed**

Ln 202 – add “the” before “post-rescue”

**AR: added**

Ln 205 – add ‘remained attached’ after ‘net’

**AR: added**

Fig 5 legend or Y-axis must state what is the number – ie “breaths/minute”

**AR: added in the legend**

Ln 219 – add ‘bin’ after ‘minute’.

**AR: added**

Your statistic treats each one minute bin as an independent data sample, leading to a high sample size of measurements. Therefore, you need to be clear that you are comparing breaths per BIN specifically.

**AR: thank you for the suggestion, we added ‘bin’ after ‘minute’**

Ln 238 – change ‘are’ to ‘were’

**AR: changed**

Ln 241 – Delete “The first part of dataset included 56 minutes of acoustic recordings that were analysed for” and add ‘were analysed’ after “(18 July)”

**AR: delete the first phrase and added the second**

Ln 244 change ‘differed in’ to ‘differed between’

**AR: changed**

Ln 254 – delete ‘Whereas, ‘

**AR: done**

Ln 259 – change “The second part of dataset included 1519 minutes (from 19:04 to 06:15) of acoustic recordings of second whale’ re-sighting on 5th October.” To “An additional 1519 minutes (from 19:04 to 06:15) of acoustic recordings were recorded during the second whale’s re-sighting on 5th October.”

**AR: changed**

Ln 272 – state the sample size for this KW test.

**AR: added**

Ln 274 – change ‘first one’ to ‘first whale’

**AR: changed**

Ln 282 – change ‘has been’ to ‘were’

**AR: changed**

Ln 293 – change ‘higher trains duration’ to ‘higher train duration’

**AR: changed**

Lns 294-297. Specify the samples sizes used for all statistical tests.

**AR: Done**

Ln 303 – change ‘observed’ to ‘noted’

**AR: changed**

Ln 316 – change ‘smaller’ to ‘lower’

**AR: changed**

Fig 9 legend – Change “Coda types spectrograms” to “Spectrograms of coda types”

**AR: changed**

Ln 326 – change “3-clicks” to “3-click”

**AR: changed**

Ln 331 – change ‘a stressed’ to ‘active’

**AR: changed**

Ln 336 – change “pattern, especially on acoustic’ to “patterns, including acoustic”

**AR: changed**

Ln 338 – change “outputs” to “resulting observations”

**AR: changed**

Ln 345 – change “brought to free” to “successfully freed”

**AR: changed**

Ln 347 – change ‘run away’ to ‘swim away’

**AR: changed**

Ln 348 change “whales’s” to “whales’”

**AR: changed**

Ln 363 – add “The” before ‘First whale”

**AR: added**

Ln 364 – add “The” before “second whale”, and add “the” before “first” on the same line

**AR: added**

Ln 365 – change “to the hypothesis” to “with the hypothesis”

**AR: changed**

Ln 386 – change “resembling” to “resembled”

**AR: changed**

Ln 387 – change “a foraging behaviour” to “echolocation-based foraging”

**AR: changed**

Ln 389 – change ‘are very low’ to ‘were very low’

**AR: changed**

Ln 394 – change “are used” to “were used”

**AR: changed**

Ln 395 – delete “eventual”

**AR: deleted**

Ln 396 – change “do not fit” to “did not fit”

**AR: changed**

Ln 399 – change ‘in accordance with the stress level’ to ‘in accordance with the activity level’

**AR: changed**

Ln 401 -change “triggered by high-stress” to “during high activity”

**AR: changed**

Ln 409 – change “sight” to “encounter” both times.

**AR: changed both**

Ln 414 – use past tense. Change “are” to “were” and “share” to “shared”

**AR: changed**

Ln 415 – change “are” to “were”

**AR: changed**

Ln 416 – change “stressful” to “entanglement”

**AR: changed**

Ln 418 – change ‘are definitely’ to ‘were definitely’

**AR: changed**

Ln 421 – change ‘show’ to ‘had’

**AR: changed**

Ln 423 – add “were” before “operating”

**AR: added**

Ln 425 – change “to high” to “with high”

**AR: changed**

Ln 434 – change ‘enough’ to ‘sufficient’ and change “meaning” to “function”

**AR: changed**

Ln 437 – change “have known” to “are known”

**AR: changed**

Ln 449 – change “are” to “were”

**AR: changed**

Ln 453 – change “are more” to “were more”

**AR: changed**

Ln 460 – change “Orchinus” to “Orcinus”

**AR: changed**

Ln 461 – change “heard” to “produced”

**AR: changed**

Ln 474 – I don’t think this is the first, nor is it important if it is the first or not. See Esch et al 2009 Whistles as Potential Indicators of Stress in Bottlenose Dolphins. Journal of Mammalogy.

**AR: We agreed with your correction and we changed ‘is a first attempt’ with ‘is proposing’.**

Ln 484 – delete “several” before “boats”

**AR: deleted**

Ln 508 – change to “Acknowledgements”

**AR: changed**

Ln 518 – change “any ethics statement is” to “no ethics statement is required”

**AR: changed**
